# The prognostic influence of intrapancreatic tumor location on survival after resection of pancreatic ductal adenocarcinoma

**DOI:** 10.1186/s12893-015-0110-5

**Published:** 2015-11-28

**Authors:** Dietrich A. Ruess, Frank Makowiec, Sophia Chikhladze, Olivia Sick, Hartwig Riediger, Ulrich T. Hopt, Uwe A. Wittel

**Affiliations:** Department of Surgery, University of Freiburg, Freiburg, Germany; Department of Surgery, Vivantes-Humboldt-Clinic, Berlin, Germany

**Keywords:** Pancreatic cancer, Pancreatoduodenectomy, Distal pancreatectomy, Survival, Outcome

## Abstract

**Background:**

The prognosis of pancreatic ductal adenocarcinoma (PDAC) is worse when the tumor is located in the pancreatic body or tail, compared to being located in the pancreatic head. However, for localized, resectable tumors survival seems to be at least similar.

**Methods:**

We analyzed and compared the outcome after pancreatoduodenectomy (PD) and distal pancreatectomy (DP) for PDAC at our institution. Clinical, pathological and survival data from patients undergoing pancreatic resection for PDAC 1994–2014 were explored retrospectively, accessing a prospective pancreatic database. Patients receiving primary total pancreatectomy were excluded.

**Results:**

Four hundred and thirteen patients were treated for PDAC: 347 (84 %) underwent PD and 66 (16 %) DP. Tumors located in the pancreatic body and tail were significantly larger than their counterparts located in the head (30.6 mm vs. 41.2 mm; *p* < 0.001). However, distal tumors had significantly less nodal involvement (71 % vs. 57 %; *p* = 0.03). Portal-vein resection (PVR) was performed more often in PD, multivisceral resection (MVR) was more frequent in DP (37 % vs. 14 % and 4 % vs. 29 %; *p* < 0.001). Rates for negative resection margins and tumor grading were similar. Postoperative complication rates including morbidity, rates of re-operation and mortality were comparable. Long-term outcome revealed no significant difference between PD and DP with 5-year survival rates of 17.8 and 22 % respectively (*p* = 0.284). Multivariate analysis confirmed positive resection margin, positive nodal status, extended resection (PVR, MVR) and lack of adjuvant/additive chemotherapy as independent risk factors for poor survival after pancreatic resection.

**Conclusion:**

Patients with resectable pancreatic ductal adenocarcinoma located in the body and tail of the pancreas display a similar postoperative oncological outcome despite larger tumors when compared to patients with resectable tumors located in the pancreatic head.

## Background

Prognosis for pancreatic cancer (PC) has only slightly improved over the past decades and still is grim, with a current 5-year relative survival rate of about 6.9 % [1, 2]. Stratified by tumor site, mortality is even worse when the tumor is located in the pancreatic body and tail. A recent analysis based on SEER-data (*Surveillance, Epidemiology and End Results Program* by the National Cancer Institute, NCI, U.S.) revealed a significant difference in the 3-year survival rates of 3.9 % (body/tail) vs. 6.2 % (head) [3]. This observation is most likely due to delayed diagnosis in the case of tumor location in the pancreatic body and tail, since early symptoms are usually lacking. Therefore, a significantly higher percentage of patients is primarily diagnosed with advanced disease and stage IV PC (body/tail: 56–73 % vs. head: 26–39 %) [3, 4], where surgical therapy is not beneficial. Complete resection at an early stage though, is the most important factor in multimodal treatment and the only chance for cure and long-term survival [5]. In combination with adjuvant chemotherapy, 5-year survival rates of up to 15–30 % can be achieved [6–9].

In the case of localized and resectable disease, some observational studies analyzing the outcome by tumor stage at diagnosis show superior survival for patients with cancer located in the pancreatic body/tail compared to patients with cancer located in the pancreatic head [3, 4]. A NCD-report (*National Cancer Database*, American College of Surgeons Commission on Cancer and American Cancer Society) pictures a 5-year survival-rate of 32 % (tail) vs. 11 % (head) for stage I disease (for stage II and stage III disease 12 % (tail) vs. 6 % (head) and 11 % (tail) vs. 7 % (head), respectively) [4]. However, when recently a surgical collective of patients after pancreatic resection for adenocarcinoma was examined, the survival benefit for patients with resectable disease located in the body and tail detected in these observational studies could yet not be confirmed [10]. In this and other studies, despite significantly larger size of tumors located in the body/tail, survival after proximal pancreatectomy and distal pancreatectomy was similar [6, 10–13].

To clarify these putative contradictions, our aim was to retrospectively examine our own single-institution collective regarding survival after resection for pancreatic ductal adenocarcinoma (PDAC). We intended to identify risk factors influencing survival in patients undergoing distal pancreatectomy (DP) or pancreatoduodenectomy (PD) for PDAC.

## Methods

### Patients

From July 1994 to December 2014, 413 patients with primary non-metastasized pancreatic adenocarcinoma were eligible for surgery (PD or DP) in our department (patients requiring total pancreatectomy were not included). 347 were treated by pancreatoduodenectomy and 66 by distal pancreatectomy.

### Surgical technique and pathological analysis

Whenever possible, the pylorus was preserved during pancreatoduodenectomy. Complete lymphadenectomy was performed in the hepatic ligament, right of the celiac trunk and right of the mesenteric artery. Venous structures were resected whenever necessary to achieve complete resection. Intraoperative histopathologic evaluation was routinely performed at common bile duct and pancreatic resection margin. In recent years the mesopancreatic retroperitoneal margin was also examined. Other margins underwent frozen section analysis if intraoperatively indicated, such as in case of venous resection at both venous resection margins. In distal pancreatectomy with splenectomy, lymphadenectomy was performed left of the celiac trunk and left of the mesenteric artery. Since 2005 resection was performed as described by Strasberg et al. [8, 14, 15] including the fascia of Gerota and when indicated the left adrenal gland. Intraoperative histopathologic evaluation was performed at the pancreatic resection margin. After formalin fixation, standard histopathological evaluation was performed on all operative specimens in which tumor size, lymph node status and resection margin were assessed. Negative resection margin was defined as tumor remote to the resection margin independent of the exact distance.

Since 2006 laparoscopic procedures for DP as well as PD increased in number. Pancreatic stump management (DP) and reconstruction (PD) were accomplished via mini-laparotomy.

### Perioperative therapy

Neoadjuvant therapy was administered, in a few cases (*n* = 22) with locally advanced disease since year 2000, predominantly as radiochemotherapy. Postoperative adjuvant treatment was heterogeneous. In the early study phase adjuvant therapy was not routinely performed after curative resection. Since 2003 a few selected patients were included in studies. Later, due to evidence from randomized trials (*European Study Group for Pancreatic Cancer*: ESPAC-1 Trial) [16], adjuvant chemotherapy was regularly recommended and administered. In case of positive resection margins postoperative chemoradiation or additive chemotherapy was administered whenever applicable, preferentially in clinical trials.

### Follow-up and statistical analysis

Perioperative data was collected prospectively in a SPSS database (IBM Corp. Released 2013. IBM SPSS Statistics for Windows, Version 22.0. Armonk, NY: IBM Corp.) The survival status was achieved from general practitioners or oncologists (until 2001) and regional cancer registries (since 2001). Data collection and analysis were performed in accordance with the Helsinki guidelines and approved by the local ethics committee (*Ethik-Kommission of the Albert-Ludwigs-Universtität Freiburg*), the need for individual verbal or written informed consent from participants or their next of kin was waived. Statistical analysis was performed with SPSS. In addition to descriptive statistics, inferential analysis (*χ*^*2*^*-test for categorical variables or Mann–Whitney-U-test for continuous variables)* and Kaplan-Meier survival analyses with log-rank-test for the comparison of subgroups, multivariate analysis (Cox proportional hazards model) was performed to determine independent risk factors.

## Results

Of the 413 resected patients nine (2.2 %) died due to postoperative complications, seven were lost to follow-up. Therefore, 16 patients were excluded for survival analysis (11 patients from the PD-group and 5 patients from the DP-group). Survival was analyzed in the remaining 397 patients. Of those, 336 patients received PD while 61 patients were treated with DP. Median postoperative follow-up was 14 months (13 months for deceased patients; 16 months for censored patients).

### Patient characteristics

In both groups patient characteristics were not significantly different in regard to gender, age and body mass index (Table 1).

**Table 1:** Demographic, surgical, pathological and postoperative data from 413 patients undergoing pancreatic resection for pancreatic ductal adenocarcinoma (1994–2014)

	Pancreaticoduodenectomy	Distal pancreatectomy	*p*
N of resections	347	66	
Gender			0.755
Male	182 [52 %]	36 [55 %]	
Female	165 [48 %]	30 [45 %]	
Age in years median (range)	67.0 (31–89)	65.6 (35–88)	0.362
BMI in kg/m^2^ median (range)	24.4 (7.6–38.8)	23.6 (18.2–35.4)	0.725
Operation time in min median (range)	440 (245–760)	301 (140–717)	<0.001
PRBC received if received, vol. in ml mean (±SD)	135 [39 %]	17 [27 %]	0.056
	423 (±729)	192 (±368)	0.024
Extended resection			
- none	204 [59 %]	38 [58 %]	<0.001
- portal vein	128 [37 %]	9 [14 %]	
- multivisceral	15 [4 %]	19 [29 %]	
Free resection margin	247 [71 %]	46 [73 %]	0.767
Grading^a^			
G1	11 [3 %]	5 [9 %]	0.229
G2	194 [57 %]	28 [51 %]	
G3	128 [38 %]	21 [38 %]	
G4	6 [2 %]	1 [2 %]	
Tumor size in mm median (range)	28 (1–130)	38 (5–110)	<0.001
Node positive	246 [71 %]	35 [57 %]	0.030
N of analyzed nodes median (range)	15 (2–47)	15 (2–32)	0.783
Any complication	181 [52 %]	32 [49 %]	0.584
Surgical complication	123 [35 %]	20 [30 %]	0.421
- POPF	48 [14 %]	25 [38 %]	<0.001
- IAA	25 [7 %]	8 [12 %]	0.177
- SSI	44 [13 %]	6 [9 %]	0.413
Re-operation	33 [10 %]	10 [15 %]	0.169
Mortality	7 [2 %]	2 [3 %]	0.605
Adjuvant/additive chemotherapy	188 [54 %]	35 [53 %]	0.864

*BMI* body mass index, *PRBC* packed red blood cells, *POPF* postoperative pancreatic fistula, *IAA* intraabdominal abscess, *SSI* surgical site infection

^a^Data for Tumor grading was not available for 19 patients (PD: 8 patients; DP: 11 patients)

### Surgery

Regarding duration of surgery, a significant difference was detectable with PDs showing to be more time-consuming (PD: 440 (245–760) min vs. DP: 301 (140–717) min [median (range)]). If blood transfusions were necessary, the volume administered was significantly higher in the PD-group (PD: 423 ± 729 ml vs. DP: 192 ± 368 ml [mean ± SD]). The intraoperative involvement of the superior mesenteric vein or portal vein with a subsequent resection was higher in patients with tumors located in the pancreatic head (PD: 37 % vs. DP: 14 %). In contrast, multivisceral resections were performed more frequently in patients with tumors located in the pancreatic body and tail (PD: 4 % vs. DP: 29 %). Here atypical gastric resections and colon resections were mostly performed (data not shown). Resection of the left adrenal gland was considered as frequent part of the procedure for DP, therefore adrenalectomy was neglected when analyzing for multivisceral resections (Table 1).

### Pathologic diagnosis

Tumors located in the pancreatic body and tail were significantly larger than tumors located in the pancreatic head (PD: 28 (1–130) mm vs. DP: 38 (5–110) mm [median (range)]. Despite larger tumors, margin negative (R0) resection was achieved with an equal rate (PD: 71 % vs. DP: 73 %). Histopathologic evaluation showed a comparable distribution of tumor grading. However, nodal involvement was less frequent when the tumor was located in the pancreatic body/tail (rate of N+: PD: 71 % vs. DP: 57 %, with similar median number of analyzed nodes) (Table 1).

### Postoperative course

Postoperative complications of any kind and surgery related complications like pancreatic fistula, intraabdominal abscess, or wound infection were not significantly different between the two groups. However, evaluation by specific surgical complication revealed significance for a higher pancreatic fistula rate after distal resection. Necessity of re-operation and mortality (PD: 2 % vs. DP: 3 %) was similar (Table 1).

### Adjuvant/additive therapy

Two-hundred and twenty-three patients received or were referred to oncologists to receive postoperative chemotherapy. Twenty-two of these had additionally been treated with neoadjuvant therapy for locally advanced, initially unresectable tumors; 62 had positive resection margins. The remaining group of 175 comprised patients who were either not treated in an adjuvant/additive manner (historical cohort or due to morbidity) or for whom this data was lacking. The rate of adjuvantly/additively treated patients did not differ between the PD- (54 %) and DP-group (53 %) (Table 1).

### Survival

The entire group of 397 patients showed an overall 3- and 5-year survival after pancreatic resection of 29.5 and 18.3 % respectively with a median survival of 20.6 months (95 % CI: 17.4–23.8).

**Fig. 1:**
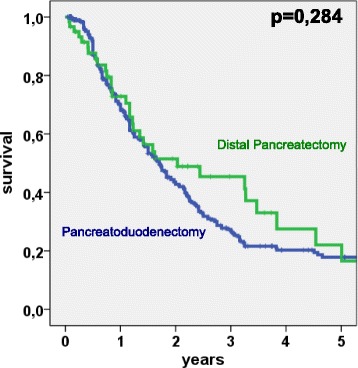
Kaplan-Meier plot: Survival analysis of 397 patients after pancreatic resection for pancreatic ductal adenocarcinoma. Patients after pancreatoduodenectomy (*n* = 336) vs. patients after distal pancreatectomy (*n* = 61). 3-year and 5-year survival rates are 27,3 and 17.8 % (PD) vs. 45.4 and 22 % (DP). *p* = 0.284

A trend to increased survival (*p* = 0.284) was observed in patients after surgery for tumors located in the pancreatic body and tail compared to those located in the pancreatic head. The 3- and 5-year survival rates of patients after pancreatoduodenectomy were 27.3 and 17.8 % compared to 45.5 and 22 % in patients treated with distal pancreatectomy. The median survival in patients after PD and DP was 20.4 months (95 % CI: 17.4–23.8) and 24.4 months (95 % CI: 2.9–45.8), respectively (Table 2 and Fig. 1).

**Table 2:** Overall survival after resection for pancreatic ductal adenocarcinoma. Univariate survival analysis of 397 patients

Parameter	*n*	3-year-survival	5-year-survival	*p*
All	397	29.5 %	18.3 %	
Tumor location				
Head	336	27.3 %	17.8 %	
Distal	61	45.4 %	22.0 %	0.284
Gender				
Male	190	29.3 %	15.8 %	
Female	207	29.7 %	20.2 %	0.683
Age				
> 65 years	225	30.4 %	22.6 %	
< 65 years	172	28.3 %	13.8 %	0.493
BMI^a^				
> 25 kg/m^2^	161	26.3 %	13.7 %	
< 25 kg/m^2^	235	31.2 %	20.9 %	0.494
Tumor size^a^				
< 30 mm	223	28.8 %	18.4 %	
> 30 mm	157	30.4 %	16.9 %	0.860
Tumor grading^a^				
Grade 1 + 2	230	29.6 %	18.7 %	
Grade 3 + 4	151	26.5 %	15.3 %	0.191
Resection margin^a^				
R0	283	36.2 %	21.7 %	
R+	113	13.5 %	9.9 %	<0.001
Nodal status^a^				
Negative	121	41.1 %	27.7 %	
Positive	272	22.9 %	13.0 %	0.001
Extended resection				
None	233	35.9 %	26.1 %	
Portal Vein (PV)	133	21.5 %	7.9 %	
More than PV, multivisceral	31	12.8 %	0 %	<0.001
PRBC transfusion^a^				
Yes	143	22.4 %	15.6 %	
No	252	33.8 %	19.2 %	0.005
Any complication				
Yes	200	31.1 %	17.2 %	
No	197	27.8 %	18.7 %	0.916
Surgical complication				
Yes	133	37.0 %	18.1 %	
No	264	25.9 %	17.4 %	0.149
Adjuvant/additive chemotherapy				
Yes/Intended^b^	222	37.3 %	14.9 %	
No/Unknown	175	22.0 %	15.7 %	0.010

*BMI* body mass index, *PRBC* packed red blood cells

^a^Some parameters were not available for individual patients (BMI: 1 patient; Tumor size: 17 patients; Tumor grading: 16 patients; Resection margin: 1 patient; Nodal status: 4 patients; PRBC transfusion: 2 patients)

^b^Included are 22 patients with (additional) neoadjuvant therapy and 62 patients with additive therapy

**Fig. 2:**
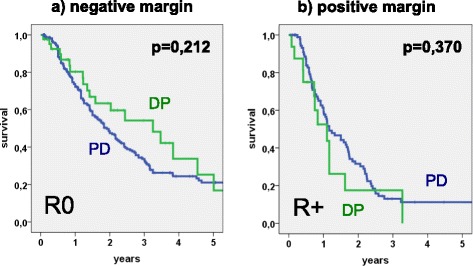
Kaplan-Meier plot: Survival analysis stratified by resection margins. **a** Patients with negative resection margin (R0), after pancreatoduodenectomy (PD, *n* = 239) vs. distal pancreatectomy (DP, *n* = 44). **b** Patients with positive resection margin (R+), after PD (*n* = 97) vs. DP (*n* = 16)

In the subgroups with negative resection margin 3-year and 5-year survival rates were 33.7 and 21.1 % (PD) vs. 54.2 and 25.3 % (DP), respectively (*p* = 0.212). In the case of margin-positive resection, survival was considerably worse with 3-year and 5-year survival rates of 13 and 11.2 % after PD vs. 17.5 % vs. 0 % after DP (*p* = 0.370) (Fig. 2).

The *Strasberg*-approach for distal resection was performed since 2005 on 20 out of 61 patients with follow-up data. We could not detect a difference in survival between the historical and the *Strasberg*-cohort (data not shown).

**Fig. 3:**
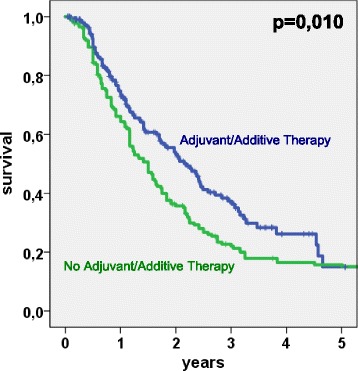
Kaplan-Meier plot: Survival analysis of 397 patients after pancreatic resection for pancreatic ductal adenocarcinoma. Patients who received or intended to receive adjuvant/additive chemotherapy (*n* = 222) vs. patients who did *not* receive adjuvant/additive therapy or with unknown status (*n* = 175)

Adjuvant/additive chemotherapy significantly improved oncological outcome after pancreatic surgery for PDAC. The group of patients who received or intended to receive adjuvant/additive therapy demonstrated a median survival of 25.8 months (95 % CI: 20.6–31.0). However, patients who did not receive adjuvant/additive therapy or for whom this information was lacking had a median survival of only 18 months (95 % CI: 14.9–21.1). 3-year and 5-year survival rates were 37.3 and 14.9 % (received/intended to receive) vs. 22 % and 15.7 % (not received/unknown), respectively (*p* = 0.010) (Table 2 and Fig. 3).

### Risk factors for survival

In *univariate analysis* resection margin, nodal disease, extent of resection (portal vein and/or multivisceral), blood transfusion and adjuvant/additive chemotherapy showed significant impact on survival after pancreatic resection for PDAC. No effect was observed for patient gender, age and BMI. Furthermore, tumor size, tumor grading and the presence of postoperative complications also did not significantly affect survival (Table 2).

*Multivariate survival analysis* revealed resection margin, nodal disease, extended resection, and adjuvant/additive chemotherapy as independent risk factors for survival after pancreatic resection for PDAC (Table 3).

**Table 3:** Multivariate analysis. Independent risk-factors for poor survival after pancreatic resection (pancreatoduodenectomies *and* distal pancreatectomies) for pancreatic ductal adenocarcinoma

	*P*-value	RR	95 %-CI
Positive resection margin	<0.001	1.7	1.3–2.2
Positive nodal status	<0.01	1.5	1.1–2.0
Extended resection			
- none	-	1	
- Portal Vein (PV)	<0.001	2.7	1.7–4.3
- more than PV (multivisceral)	<0.005	2.2	1.4–3.6
No adjuvant/additive chemotherapy (or unknown)	<0.05	1.4	1.1–1.8

## Discussion

Although stage-independent overall survival is worse for distally located pancreatic cancer, better long-term outcome for localized tumors of the pancreatic body/tail has been reported. This is probably due to surgical approaches. Delayed diagnosis of pancreatic cancer, when the tumor is located in the pancreatic body or tail, leads to a higher number of patients not being amenable to resection. Especially the lack of specific symptoms such as jaundice is responsible for that fact. Most likely, decreased survival is rather due to systemic spread and metastasis than to local unresectability. When located in the pancreatic body and tail, compared to the pancreatic head, larger tumors can be resected more frequently and successfully, as measured by the rate of margin-free resections. In spite of larger tumor size, similar long-term outcome for DP and PD has been noted [6, 10–13].

One reason for this might be a favorable tumor biology of resectable PDAC of the pancreatic body/tail. Although the tumor grade usually increases with tumor size [6], this could not be demonstrated in our analysis. However, others have reported similar data with proximal tumors showing more dedifferentiation in spite of smaller size [17]. Similar to our study, nodal involvement seems to be less frequent in resectable distal tumors [6, 17]. Confirmed here as independent risk factor, nodal disease is well known to negatively correlate with survival [18–23].

Furthermore, technical advances in form of the radical antegrade modular pancreatosplenectomy, described by Strasberg et al. [14], may achieve higher rates of margin-free resection. This has probably contributed to improved survival-rates after DP in recent years [24]. Five-year-survival-rates of up to 30 % are reported [8, 21]. However, in our cohort we could not detect a survival benefit related to this procedure.

Another factor is the possibility of resecting adjacent organs when involved in the tumor. Patients with tumors located in the pancreatic body and tail benefit from the fact that technically, distal pancreatectomies can be easily performed together with en bloc gastric or colon resections. Without the involvement of vital structures as in the case of the pancreatic head with its anatomical proximity to important vessels, this extended, or multivisceral resection is not only feasible but also safe and justified [25–27]. Our pooled data for PD and DP demonstrate that survival after portal vein (PVR) and especially multivisceral (MVR) resection is worse in contrast to the standard procedure. However, this observation may be biased by the skewed dataset in favor of PDs where MVR or extended resection for pancreatic head cancer is associated with increased perioperative risk and with worse oncological outcome [28]. In our study significantly more patients were able to undergo multivisceral resections when presenting with tumors of the pancreatic body/tail. Morbidity and mortality were not different between the two groups. Rates of margin-free resection were similar. As our data undermine (Table 2+3, Fig. 2), R0-resection is the mainstay of surgical therapy and one of the most important factors influencing long-term outcome [6, 17, 19, 29, 30]. However, it is to be completed by adjuvant chemotherapy (Table 2+3, Fig. 3) whenever possible [9].

Although this study is limited by its retrospective character, in summary our collective demonstrates similar postoperative oncological outcome for patients with resectable pancreatic ductal adenocarcinoma located in the body/tail or head of the pancreas. Therefore, the overall poorer survival in patients with tumors located in the pancreatic body or tail appears to be solely due to delayed diagnosis of disease with advanced stage and limited therapeutic options.

## Conclusion

Patients with resectable pancreatic ductal adenocarcinoma located in the body and tail of the pancreas display a similar postoperative oncological outcome despite larger tumors when compared to patients with resectable tumors located in the pancreatic head.
